# A single residue affects the dynamics and shape of a tetrameric GH43 β‐1,4‐d‐xylosidase from *Levilactobacillus brevis* DSM1269

**DOI:** 10.1002/pro.70299

**Published:** 2025-09-13

**Authors:** Javier A. Linares‐Pastén, Reza Faryar, Sergio Torrez Alvarez, Khalil Albasri, Bashar Shuoker, Maher Abou Hachem, Derek T. Logan, Eva Nordberg Karlsson

**Affiliations:** ^1^ Biotechnology and Applied Microbiology, Department of Process and Life Sciences Engineering, LTH Lund University Lund Sweden; ^2^ Instituto de Investigaciones Químicas IIQ Universidad Mayor de San Andrés UMSA La Paz Bolivia; ^3^ Department of Biotechnology and Biomedicine Technical University of Denmark Kongens Lyngby Denmark; ^4^ Biochemistry and Structural Biology, Centre for Molecular Protein Science, Department of Chemistry Lund University Lund Sweden

**Keywords:** 3D‐structure, glycoside hydrolase family 43, oligomeric state, xylosidase

## Abstract

Lignocellulosic materials (e.g., straw and bran) are gaining interest as feedstocks for the manufacture of higher value products, recognizing xylooligosaccharides (XOS) as interesting prebiotic compounds, and putting enzymes converting xylooligosaccharides into focus. In this work, we are investigating a XOS converting enzyme from the probiotic bacterium *Levilactobacillus brevis* (formerly *Lactobacillus brevis*). Growth of multiple *L. brevis* strains, recognized as probiotics, is promoted by the presence of XOS. This study elucidates the 3D structure of an intracellular *L. brevis* β‐xylosidase, *Lb*Xyn43B from glycoside hydrolase family 43 (GH43), resolving it to 1.9 Å resolution. Functional analysis identified *Lb*Xyn43B as a mediator for XOS utilization in *L. brevis* DSM1269. The enzyme, featuring a 5‐fold β‐propeller fold typical of GH43, prefers short XOS substrates, notably β‐1,4‐xylobiose and β‐1,4‐xylotriose, aligning with its role in hydrolyzing internalized XOS. Intriguingly, crystallographic and size exclusion chromatographic evidence reveals *Lb*Xyn43B to be a tetramer, contrary to the dimeric structure previously reported in a closely related homolog from strain DSM20054. Despite sharing a high sequence identity (differing in only five residues), Thr274 in *Lb*Xyn43B within a subunit interface loop was found to influence the tetramer's shape. When mutated to Ala (the residue at the corresponding position in the homolog), the enzyme's apparent native molecular mass was impacted, resulting in a more compact oligomeric structure of slightly higher thermostability. Kinetic analysis and molecular dynamics simulations further suggest an effect of Thr274Ala substitution in modulating the accessibility of longer oligosaccharides, such as β‐1,4‐d‐xylotetraose, to the active site.

## 1 INTRODUCTION

Xylans, the heterogeneous polysaccharides found in the secondary cell wall of lignocellulosic plant biomass, are one of the most abundant types of hemicellulose. Xylans are composed of a backbone of β‐1,4‐linked xylose units that, dependending on the origin of the xylans, are decorated with different types of substituents, such as arabinose, uronic acids, acetyl, and feruloyl groups (Peña et al., 2016).

The need to harness and valorize the residual lignocellulosic biomass generated by industrial agriculture has put a focus on the utilization of different by‐products or underutilized lignocellulosic materials (e.g., straw and bran) as feedstocks for the manufacture of higher value products. With this perspective, the transformation of xylan into oligosaccharides with prebiotic properties is gaining interest (Linares‐Pasten et al., 2018; Nordberg et al., 2018).

Xylooligosaccharides (XOS) are recognized as prebiotic compounds, as they selectively stimulate the growth of beneficial bacteria (probiotics) in the human gastrointestinal tract, thereby contributing to improved gut health (Linares‐Pastén et al., 2025). Many bacterial species proposed as probiotics are classified under the genera *Bifidobacterium* and *Levilactobacillus* (Picard et al., 2005; Wang et al., 2007). Neither *Levilactobacillus* nor most *Bifidobacterium* species can degrade polymeric xylan, and to promote their growth, pre‐hydrolysis of the xylan polymers to XOS is necessary. On the other hand, species from the orders Bacteroidales (e.g., *Bacteroides xylanisolvens* (Despres et al., 2016), *Prevotella copri* (Linares‐Pastén, Hero, et al., 2021)) and Lachnospirales (e.g., *Roseburia intestinales* (Leth et al., 2018)) are important in the gastrointestinal tract. They can convert xylan into XOS, which in turn can be used by species from the genera *Levilactobacillus* and *Bifidobacterium* present in the gastrointestinal ecosystem.

*Levilactobacillus brevis* includes several strains with potential as probiotics. *In vitro* studies have revealed that *L. brevis* is one of the best XOS fermenters among lactic acid bacteria (LAB) (Crittenden et al., 2002). Indeed, our previous studies have affirmed that *L. brevis* DSM1269 utilizes XOS from several xylan sources, including wheat straw (Faryar et al., 2015), wheat bran (Immerzeel et al., 2014), rye flour, and birch wood (Falck et al., 2013).

Xylosidases of GH43 from other LAB species have been identified as candidate enzymes for the intracellular degradation of XOS into xylose (Falck et al., 2015), making it likely that the corresponding enzyme in *L. brevis* DSM1269 has a similar role. Moreover, the enzymes responsible for the conversion of oligosaccharides into monosaccharides, such as the *L. brevis* enzymes, have industrial potential in the conversion of the biomass to compounds that can be metabolized by other species, resulting in industrially interesting products (Michlmayr et al., 2013).

This enzyme, from strain DSM1269, shares a high sequence identity with GH43 β‐xylosidases from other *L. brevis* strains, including DSM20054 (Michlmayr et al., 2013) and ATCC367 (Jordan et al., 2013), differing at only a few amino acid positions. Despite this close homology, differences have been reported in the native oligomeric state of the enzymes, suggesting that specific non‐conserved residues may influence quaternary structure or stability. In this study, we report the biochemical characterization and high‐resolution crystal structure of the GH43 β‐xylosidase from *L. brevis* DSM1269 (*Lb*Xyn43B). Structural analysis revealed a tetrameric assembly involving loop‐mediated subunit interactions. Based on these findings, and guided by comparative structural insights, we introduced a Thr274Ala substitution at a non‐conserved interfacial loop to investigate its impact on the enzyme's quaternary structure, stability, and catalytic properties. Our results provide structural and functional insight into how a single residue can modulate enzyme shape and dynamics in a highly conserved GH43 subfamily enzyme involved in XOS metabolism.

## 2 MATERIALS AND METHODS

### 2.1 Bacterial strains, culture media, and vector

*Levilactobacillus brevis* DSM 1269 was obtained from the German Collection of Microorganisms and Cell Cultures (DSMZ). Cloning host *Escherichia coli* NovaBlue and expression strain *E. coli* BL21(DE3) were from Novagen. *L. brevis* was cultivated in Man, Rogosa, and Sharpe (MRS)‐broth (Difco) at 37°C for 24 h in anaerobically prepared vials of 100 mL. *E. coli* strains were cultivated in Luria Bertani (LB)‐broth at 37°C overnight and 30°C during the expression period of the recombinant protein. Cloning vector pUC19 was purchased from Fermentas, and expression vector pET28b(+) was purchased from Novagen. Ampicillin (100 μg/mL) was added for the selection of *E. coli* strains harboring the pUC19 construct, and kanamycin (30 μg/mL) was added for the selection of *E. coli* strains harboring the pET28b construct.

### 2.2 Cloning of the gene encoding *Lb*Xyn43B (wt)

A gene encoding a β‐xylosidase (*Lb*Xyn43B) from *L. brevis* DSM 1269 was cloned (GenBank: KX174294). *L. brevis* genomic DNA was extracted using the E.Z.N.A. Genomic Isolation Kit (Omega Bio‐Tek, USA). The putative gene encoding the βd‐xylosidase was amplified from the genomic DNA using the following primers: forward AGC CAT ATG AAG ATT CAA AAT CCA GTT TTA CCC GG and reverse GTG CTC GAG TTA ATC TGG CAG TTC TTG ATA ATC AAA GTA ATC AAA G, with restriction sites for NdoI and XhoI, respectively (underlined). These primers were designed according to the gene sequence (LVIS_2285) of the type strain *L. brevis* ATCC*®* 367*™* deposited in GenBank (accession CP000416.1) (Makarova et al., 2006). The obtained amplicon was inserted in the SmaI restriction site of pUC19, giving the construct pUC19::*Lb*Xyn43B, and propagated in *E. coli* NovaBlue. The complete sequence of the *Lb*Xyn43B‐encoding gene was obtained by sequencing the vector from both the 5′ and 3′ ends, using a pair of sequencing primers annealing in the regions M13 forward and reverse of the pUC19 vector. Later, the gene was subcloned between the restriction sites NdeI and XhoI of the expression vector, giving the construct pET28b::*Lb*Xyn43B.

### 2.3 Multiple sequence alignment (MSA)

Sequence similarity searches were made in the NCBI BLAST suite (Altschul et al., 1990) with blastp against the non‐redundant protein sequence database, Swiss‐Prot database, and PDB database using default algorithm parameters. The strucutre of *Selenomonas ruminiatum* was one of the most similar (PDB 3C2U) MSA was performed in the Jalview program using the Clustal Omega alignment tool (Waterhouse et al., 2009).

### 2.4 Prediction of the three‐dimensional structure

A model of the three‐dimensional structure was obtained using the AlphaFold3 server (https://alphafoldserver.com/) (Jumper et al., 2021; Mirdita et al., 2019; Mirdita et al., 2022). The model confidence was assessed by the pLDDT score (Jumper et al., 2021) and the top ranked model was used for subsequent analysis. The model was compared with the crystal structure (below).

### 2.5 Cloning of the variant encoding *Lb*Xyn43B/Thr274Ala

Site‐directed mutagenesis was made using a ligation‐independent technology. The subcloned construct was used as the template for obtaining a mutant with the codon corresponding to Thr274 mutated to Ala. The resulting pET28b::*Lb*Xyn43B/Thr274Ala was obtained by site‐directed mutagenesis with overlapping forward and reverse mutagenic primers for full plasmid amplification. The following primers were used: forward CGC GCG TGG AAC CAC **G**C**G** AAC GAA TCC AGT CAT G and reverse CATG ACT GGA TTC GTT **C**G**C** GTG GTT CCA CGG CCG CG, where the mutated codon (Thr274Ala) is underlined. The mutant clones were verified by DNA sequencing (Eurofins GATC, Germany).

### 2.6 Production and purification of recombinant *Lb*Xyn43B and *Lb*Xyn43B/Thr274Ala

The genes encoding *Lb*Xyn43B and *Lb*Xyn43B/Thr274Ala were expressed in BL21(DE3) and Rosetta‐gami™ 2(DE3) *E. coli* strains (Novagen). Expression was induced by the addition of isopropyl‐β‐d‐1‐thiogalactopyranoside (IPTG) at a final concentration of 1 mM when the optical density at 600 nm (OD_600nm_) of the culture reached 0.6 and was maintained for 12 h at 22°C. Cell pellets were harvested by centrifugation at 3000*g* for 10 min, and the cells were lysed using *BugBuster*® 1X Protein Extraction Reagent (Novagen). The lysed cell suspension was centrifuged to remove the cell debris.

*Lb*Xyn43B and *Lb*Xyn43B/Thr274Ala from the supernatant obtained after cell lysis were purified by immobilized‐metal ion affinity chromatography (IMAC) on an ÄKTA prime system with a HiTrap affinity column (GE Health Care, Germany). The binding buffer was 100 mM Tris–HCl and 0.5 M NaCl, pH 7.4, and the elution buffer was the same but with an additional 500 mM imidazole. Prior to further analyses, the elution buffer was exchanged by dialysis to the buffer defined under the respective section below.

Protein concentration was estimated by absorbance measurements at 280 nm wavelength, using the theoretically determined extinction coefficient, 138,200 M^−1^ cm^−1^, through ProtParam at Expasy (https://web.expasy.org/protparam/).

Productivity parameters were obtained regarding the cell density (*x*) and recombinant protein per volume of cultivation (*p*). It was estimated that 1OD_*λ*=600nm_ correspond to 0.5 g/L cell dry weight. Thus, the yield (*Y*_*p*/*x*_) and total productivity (*Q*_*p*_) were calculated with the following equations:

$$Y_{p/x}=\frac{p-p_{0}}{x-x_{0}},$$

$$Q_{p}=\frac{\left(p-p_{0}\right)}{\left(t-t_{0}\right)},$$

where *t* is the time.

### 2.7 Molecular mass of the produced proteins

The molecular masses of purified *Lb*Xyn43B and *Lb*Xyn43B/Thr274Ala were initially estimated by size exclusion chromatography as described before (Michlmayr et al., 2013). A Sephacryl S‐300 column (110 mL) (GE Healthcare) was used at a flow rate of 0.15 cm/min (0.1 mL/min) in 50 mM sodium phosphate, 150 mM NaCl, pH 7.0. Calibration was done with molecular weight standards in the range of 20–700 kDa (Sigma‐Aldrich).

Further analysis was made using a multi‐detection size exclusion chromatography system (OMNISEC). OMNISEC analyses of the oligomeric forms of *Lb*Xyn43B and *Lb*Xyn43B/Thr274Ala were performed at the LP3 facility, Lund University. The in‐solution proteins were analyzed in 50 mM sodium phosphate, pH 7, and 150 mM NaCl. For that, 50 μL of 4.5 mg/mL of sample was injected in triplicate in an OMNISEC system (Malvern Panalytical) composed of the OMNISEC RESOLVE module (integrating a Superdex 200 Increase 10/300 GL (Cytiva), with a combined pump, degasser, autosampler, and column oven) and the OMNISEC REVEAL, an integrated multi‐detector module (light scattering (RALS 90° angle and LALS 7° angle), differential refractive index, viscometer and diode‐array‐based UV/VIS spectrometer). The detectors were normalized with bovine serum albumin (Thermofisher). Data was collected and analyzed with the OMNISEC v11.41 integrated software provided by Malvern. The flow rate used was 0.5 mL/min.

Peptide mass fingerprint (PMF) analysis of the pure proteins, *Lb*Xyn43B and *Lb*Xyn43B/Thr274Ala, was performed using trypsin and ESI‐TOF mass spectrometry (MS). In addition, the mutation was confirmed by amino acid sequencing using MS.

### 2.8 Thermal stability

Thermal stability was assessed by determining the melting point (*T*_*m*_). *Lb*Xyn43B and *Lb*Xyn43B/Thr274Ala were analyzed by differential scanning fluorimetry (DSF) using intrinsic fluorescence (Choi et al., 2018; Manasian et al., 2020). The proteins were diluted to 0.5 g/L in a buffer containing 100 mM Tris–HCl and 0.5 M NaCl, pH 7.4. Subsequently, 10 μL of each sample was directly loaded into instrument capillaries. Samples were heated from 20°C to 90°C at a rate of 1°C/min. Fluorescence intensity was recorded at 330 and 350 nm. The instrument used was a Prometheus NT.48 nanoDSF (NanoTemper Technologies, GmbH, Munich, Germany), and data analysis was performed using PR control software (Version 2.0, Munich, Germany).

### 2.9 Crystallization and data collection

*Lb*Xyn43B (purified by IMAC as described above) was dialyzed against 50 mM Tris–HCl buffer pH 7.5 and concentrated to 2.5 mg/mL. Initial crystallization conditions were found using the JCSG+ and PACT Premier screens (Molecular Dimensions Ltd., UK) (Newman et al., 2005). Crystallization trials were set up in Greiner low‐profile 96‐well plates using a mosquito robot (TTP Labtech, UK) at 20°C. Drops of protein solution were mixed with reservoir solution in ratios of 100:200, 100:100, and 200:100 nL. The most promising drops were optimized with respect to PEG and salt concentration. The crystals used for data collection grew in Greiner low‐profile 96‐well plates with drops of 100 nL of protein solution and 100 nL of reservoir at 4°C. The best crystallization conditions for the native protein were 27–29% PEG 1500, 60–140 mM Malonate‐Imidazole‐Borate buffer, pH 4.0–4.5. Crystals were screened, and data were collected at 100 K at station I911‐3 of the MAX‐II synchrotron (Lund, Sweden). Diffraction data were indexed and integrated using XDS (Kabsch, 2010) and scaled using Aimless (Evans & Murshudov, 2013).

### 2.10 Structure determination and refinement

The structure was solved by molecular replacement using Phaser (McCoy et al., 2007) through the MrBUMP pipeline (Keegan & Winn, 2007) with PDB entry 3C2U (the d‐xylosidase from *Selenomonas ruminantium*), with 53.6% sequence identity, as a search model. The model was prepared using Sculptor (Bunkóczi & Read, 2011). Automatic model building was carried out using Buccaneer (Cowtan, 2006). Subsequent model rebuilding and structure refinement were performed with REFMAC5 (Murshudov et al., 2011), alternating with interactive adjustments using COOT (Emsley et al., 2010). Data processing and refinement statistics are presented in Table 2 (see Section 3).

### 2.11 Molecular dynamics simulations

The solved *Lb*Xyn43B structure and a mutated variant (*Lb*Xyn43B/Thr274Ala, modeled using the crystal structure) were subjected to molecular dynamics simulations. A tetrameric form of *Lb*Xyn43B was placed into a cubic box extending 10 Å from the protein. The solvent was simulated with explicit water molecules TIP3P, containing sodium chloride ions at a concentration of 0.9% (w/v). The temperature was set to 300 K, and the total simulation time was 300 ns. All calculations were performed in GROMACS (Abraham et al., 2015) using the AMBER03 force field (Wang et al., 2004). Periodic boundaries, 2.5 fs time steps, and 8 Å cutoff of short‐range electrostatic and van der Waals forces and long‐range forces calculated by PME were applied (Aronsson et al., 2018). The system was energy minimized using the steepest descent algorithm with a maximum of 50,000 steps, a step size of 0.1 Å, and a tolerance of 1000 kJ/mol (Bustos et al., 2020). The simulation was performed with a two‐step equilibration, NVT and NPT ensembles, 100 ps each, followed by a production phase of 270 ns (Aronsson et al., 2018; Linares‐Pastén, Jonsdottir, et al., 2021). Trajectories were saved every 1.25 ns. The root mean square deviation (RMSD) and root mean square fluctuation (RMSF) from *C*_*α*_, and radius of gyration (*r*_*g*_) were calculated. Graphical analysis and figures of molecular structures were made in Chimera (Pettersen et al., 2004). For both the wild‐type and mutant proteins, molecular dynamics were performed in triplicate, starting from different initial coordinates.

### 2.12 Kinetic characterization

Kinetic parameters were determined by measuring the activity in 50 mM sodium phosphate buffer, pH 7.0, against β‐1,4‐d‐xylobiose (X2), β‐1,4‐d‐xylotriose (X3), and β‐1,4‐d‐xylotetraose (X4), using substrate concentrations from 0.5 to 25 mM. All reactions were performed at 37°C and pH 7.0. For incubation, 2 μL of the enzyme was added to a 50 μL reaction mixture, giving a final enzyme concentration of 0.1 μg/mL. The reactions were monitored for 5 min. Reactions were stopped by incubating the samples at 95°C for 5 min, then cooling on ice for 5 min.

The hydrolysis of X2, X3, and X4 was analyzed by measuring the release of xylose using HPAEC‐PAD (DIONEX, CA) with a 250 mm × 3 mm i.d. 5.5 μm CarboPac PA200 and a guard column 50 mm × 3 mm of the same material with a mobile phase (0.5 mL/min) of constant 100 mM NaOH (Merck) and a gradient of sodium acetate (SIGMA). Values of *K*_m_ and *k*_cat_ were calculated using non‐linear regression of the Michaelis–Menten equation to the initial rate data using Excel (Microsoft Office).

### 2.13 Statistical analysis

Kinetic constants, *k*_cat_ and *K*_m_, of *Lb*Xyn43B and *Lb*Xyn43B/Thr274Ala were analyzed using an independent *t*‐test to compare the means, assuming equal variances. The significance level was set at *α* = 0.05. The statistical analysis was performed in program R 4.2.3 GUI 1.79 (www.r-project.org).

## 3 RESULTS

### 3.1 Bioinformatic analysis of the β‐d‐xylosidase *Lb*Xyn43B

The β‐d‐xylosidase *Lb*Xyn43B from *Levilactobacillus brevis* DSM1269 is a glycoside hydrolase, predicted to be involved in the intracellular degradation of xylooligosaccharides (XOS) into xylose. Such a role has previously been proposed for a related enzyme originating from the genus *Weissella* (Falck et al., 2015), and as the enzymes are homologs and both species occur in the human gut, a similar role is likely. The deduced amino acid sequence of the full‐length enzyme comprises 540 residues, excluding the 20‐residue N‐terminal His‐tag (MGSSHHHHHHSSGLVPRGSH) used for purification. This results in a calculated molecular mass of 61.4 kDa for the biological enzyme and 63.6 kDa for the recombinant variant with the His‐tag included. Signal peptide prediction using SignalP 5.0 did not result in the identification of any secretion signal, corroborating the proposed intracellular location of *Lb*Xyn43B, consistent with the proposed role in intracellular XOS metabolism.

**FIGURE 1:**
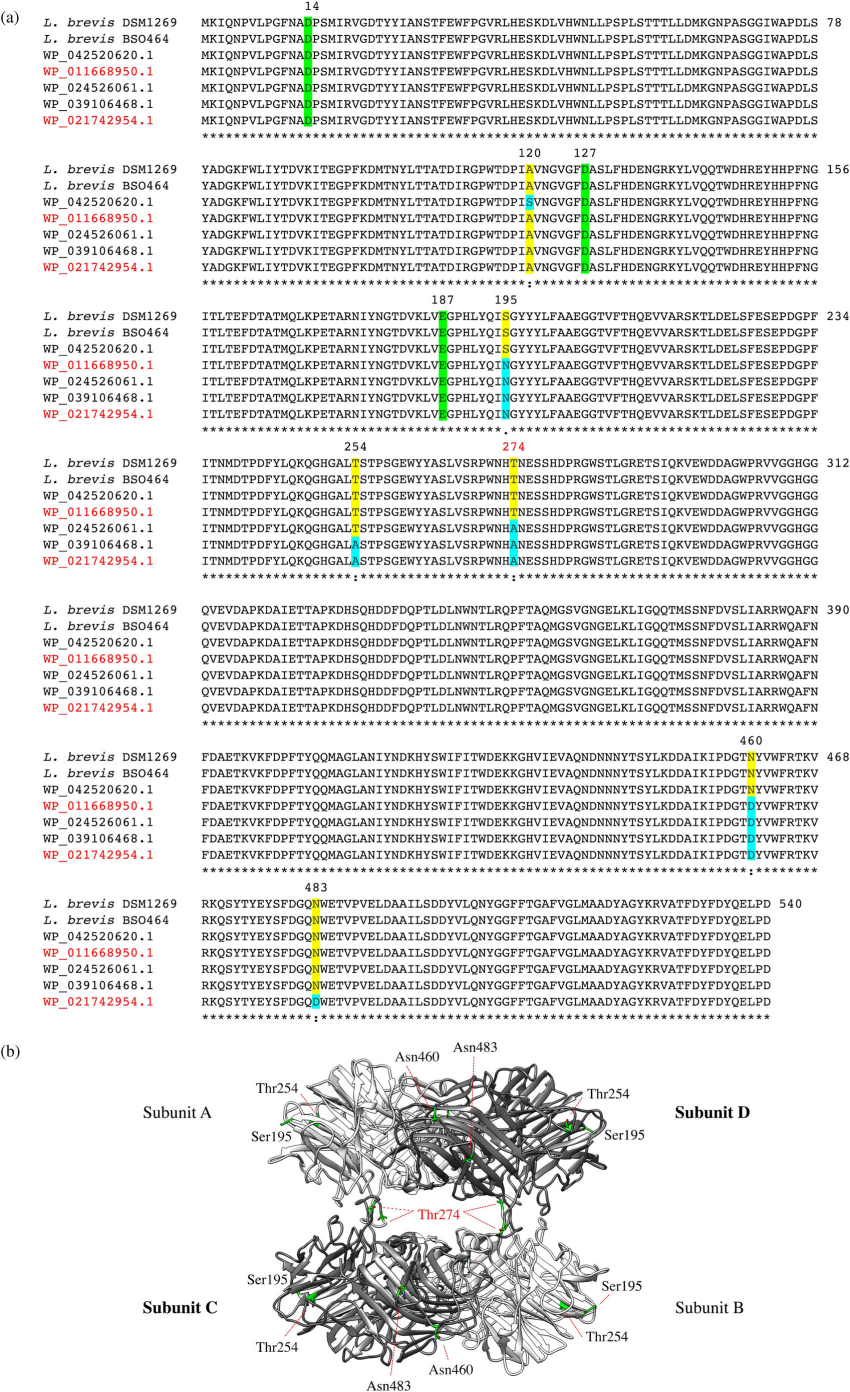
Bioinformatic analysis. (a) Multiple sequence alignment (Clustal Omega, (1.2.1)) of *β*‐xylosidases from different strains of *L. brevis*. The alignment includes: *Lb*Xyn43B from *L. brevis* DSM 1269 (studied in the present work), the xylosidase from *L. brevis* BSO464, putative enzymes encoded by deposited genes (WP_042520620.1, WP_024526061.1, WP_039106468.1) and in red sequences of LbX from *L. brevis* ATCC 367 (WP_011668950.1) and XynB2 from *L. brevis* ATCC 14869 = DSM 20054 (WP_021742954.1). Non‐conserved amino acids are in yellow and blue, including T274 of *Lb*Xyn43B, which was mutated to Ala. The catalytic triad is in green. (b) Molecular model of *Lb*Xyn43B tetramer from *L. brevis* DSM 1269, obtained with AlphaFold3.

To identify structural homologs, the *Lb*Xyn43B sequence was subjected to BLASTp analysis against the Protein Data Bank (PDB). The three closest matches with solved structures were GH43 β‐d‐xylosidases from *Selenomonas ruminantium* (PDB 3C2U, 53.6% identity), *Clostridium acetobutylicum* (PDB 1Y7B, 52.1%), and *Bacillus pumilus* (PDB 6IFE, 49.2%), all covering >99% of the *Lb*Xyn43B sequence, aiding the identification of the catalytic triad (indicated in Figure 1).

To evaluate sequence variability among related *L. brevis* strains, a multiple sequence alignment was made using Clustal Omega (Figure 1). Homologs from seven *L. brevis* strains showed a high degree of conservation within GH43 subfamily 11. The residues comprising the predicted catalytic triad were fully conserved in all aligned sequences. In fact, only six amino acid positions were found to differ across the enzymes from the *L. brevis* strains: 120 (Ala/Ser), 195 (Ser/Asn), 254 (Thr/Ala), 274 (Thr/Ala), 460 (Asn/Asp), and 483 (Asn/Asp), based on *Lb*Xyn43B numbering. Sequence comparison of the two most closely related enzymes: *Lb*Xyn43B from *L. brevis* DSM1269 (this work) and XynB2 from *L. brevis* DSM20054 (Michlmayr et al., 2013) revealed five non‐conserved residues: 195 (Ser/Asn), 254 (Thr/Ala), 460 (Asn/Asp), 483 (Asn/Asp), and 274 (Thr/Ala) (Figure 1a).

The hypothesis that one of the non‐conserved residues may affect the subunit interactions arose from a combination of comparative sequence and structural analyses. First, multiple sequence alignment of β‐xylosidases from *L. brevis* strains revealed that only a few amino acids differ among closely related homologs, with Thr274 in DSM1269 replaced by Ala in the homologs enzyme XynB2 from DSM20054. Second, structural modeling by AlphaFold3 placed Thr274 within a flexible loop at the oligomeric interface (Figure 1b), distant from the catalytic site but positioned to potentially influence inter‐subunit packing. Since XynB2 has been reported as dimeric by SEC, in contrast to the tetrameric assembly of *Lb*Xyn43B, we hypothesized that residue 274 could play a role in modulating quaternary structure, with potential downstream effects on thermostability and substrate accessibility.

### 3.2 Production and validation of wild‐type and Thr274Ala mutant

**FIGURE 2:**
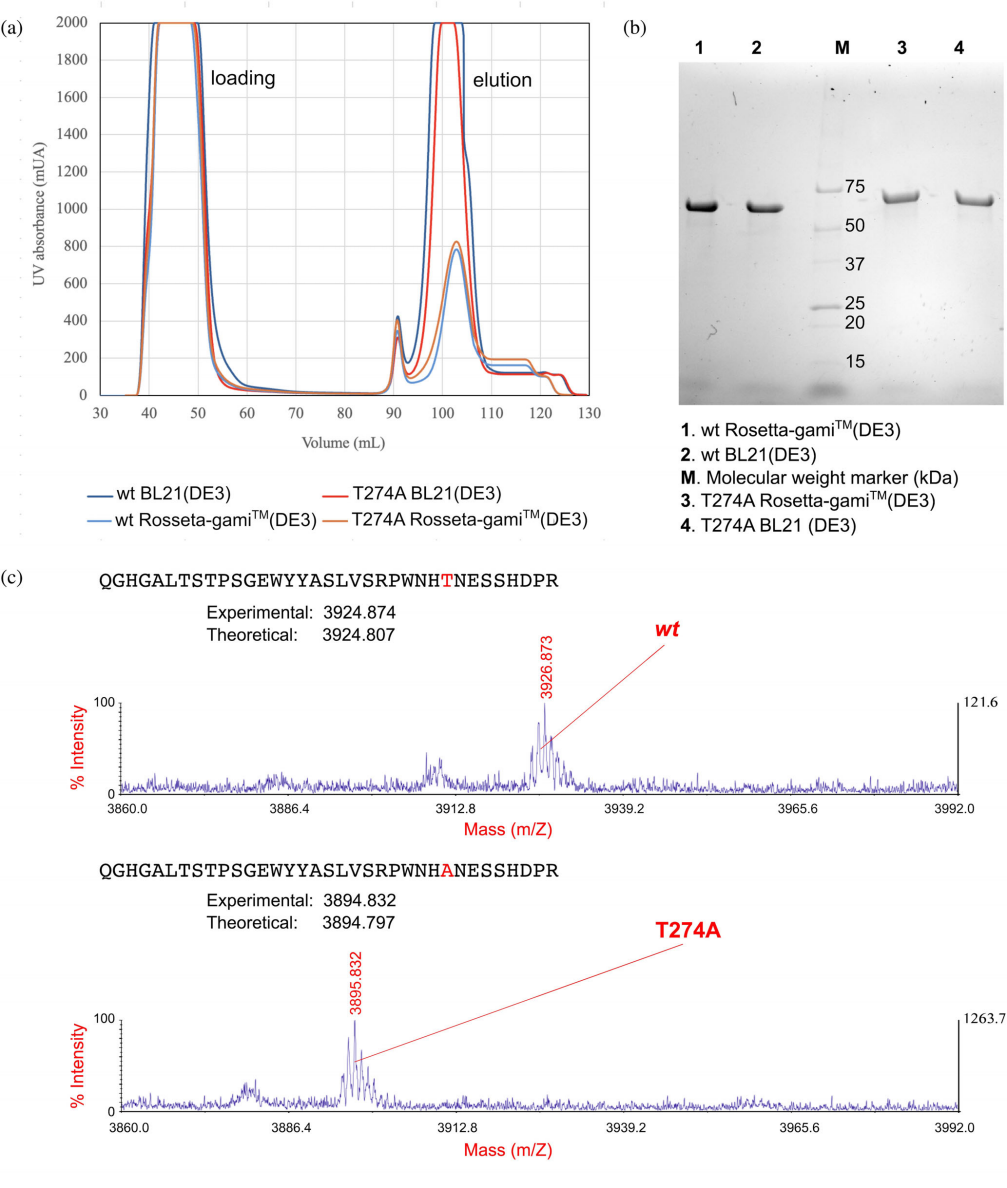
(a) Purification by IMAC of *Lb*Xyn43B and the mutant *Lb*Xyn43B/Thr274Ala from *E. coli* strains Bl21(DE3) and Rosetta‐gami™(DE3), respectively. (b) SDS‐PAGE of the produced and purified proteins. (c) Peptide mass fingerprint of purified proteins corresponding to wt and mutant Thr274Ala (T724A).

*Lb*Xyn43B from strain DSM1629 and the Thr274Ala mutated enzyme were successfully produced in *E. coli* BL21(DE3) and Rosetta‐gami™ 2(DE3), at a high production level, and purified by immobilized metal ion affinity chromatography (IMAC) to >95% purity, as estimated by SDS‐PAGE analysis (Figure 2a,b). The protein bands observed in the electrophoretic gel are consistent with the theoretically calculated molecular masses of the enzymes, including the N‐terminal His‐tag, namely 63,580.3 Da (*Lb*Xyn43B) and 63,550.2 Da (*Lb*Xyn43B/Thr274Ala), and the introduced mutation of the variant T274A was confirmed both by gene sequencing and MS analysis (Figure 2c).

The productivity of the *Lb*Xyn43B wild type (wt) and mutant *Lb*Xyn43B/Thr274A (T274A) was dependent on the selected host strain (Table 1), but both proteins were produced in high amounts in *E. coli* (Figure 2b). For both wt and T274A, strain BL21(DE3) resulted in the highest recombinant protein production, while the lowest production was observed for the wt produced in *E. coli* strain Rosetta‐gami™ 2(DE3). A smaller effect of the host strain was observed for the variant T274A. The yield coefficients (*Y*_*p*/*x*_) showed that the wt enzyme was produced equally efficiently per unit of cell mass in BL21(DE3) and Rosetta‐gami™ 2(DE3), and hence the differences in total protein production can be attributed to a higher growth rate of strain BL21(DE3) which resulted in a higher cell mass at the end of the cultivation. The yield coefficient of the variant T274A was slightly higher than the corresponding coefficient for the wt, but the specific growth rate was reduced (Table 1), with only small changes in production level per cell as a consequence of the mutation, correlating the overall production to the cell mass obtained.

**TABLE 1:** Production parameters for the recombinant enzymes *Lb*Xyn43B (wt) and *Lb*Xyn43B/Thr274Ala (T274A).

*E. coli* strain/enzyme	*x* (g/L)	*p* (g/L)	*Y*_*p*/*x*_ (g_p_ g_cell_^−1^)	*Q*_*p*_ (g_p_ h^−1^)
BL21(DE3)/wt	5.60	0.65	0.12	8.1 × 10^−3^
BL21(DE3)/T274A	2.46	0.39	0.16	4.9 × 10^−3^
Rosetta‐gami™2(DE3)/wt	1.06	0.13	0.12	1.6 × 10^−3^
Rosetta‐gami™2(DE3)/T274A	1.20	0.17	0.14	2.1 × 10^−3^

*Note*: *x* = cell density (1OD_*λ*=600nm_ correspond to 0.5 g/L cell dry weight), *p* = recombinant protein, *Y*_*p*/*x*_ = yield, and *Q*_*p*_ = total recombinant protein production.

### 3.3 Thermal stability

**FIGURE 3:**
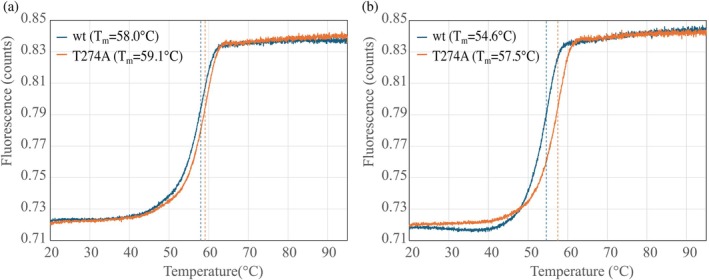
Determination of the melting points (*T*_*m*_) of *Lb*Xyn43B (wt) and *Lb*Xyn43B/Thr274Ala (T274A) by differential scanning fluorimetry. (a) Enzymes produced in *E. coli* BL21(DE3). (b) Enzymes produced in *E. coli* Rosetta‐gami™ 2(DE3).

Thermal stability analysis revealed that *Lb*Xyn43B/Thr274Ala was slightly more stable than *Lb*Xyn43B wt (Figure 3), but also that the production strain had a small influence on the thermostability. When the proteins were produced in *E. coli* BL21(DE3), the resulting melting temperature (*T*_*m*_) for the T274A mutant was 59.1°C, while for the wt, it was 58.0°C. After production in Rosetta‐gami™ 2(DE3), a *T*_*m*_ of 57.5°C was measured for the T274A mutant and 54.6°C for the wt. This shows that both the expression host and the introduced residue variation affected thermostability. The T274A mutation, however, improved thermostability regardless of the expression host. Rosetta‐gami strains are engineered to facilitate disulfide bond formation by providing an oxidative cytoplasmic environment. *Lb*Xyn43B wt and the mutant, however, lack Cys residues, and do not benefit from the oxidative cytoplasm. Conversely, the oxidative environment appears to affect their structural stability negatively, showing that for these xylosidases strain BL21(DE3) was a better production host.

### 3.4 Oligomeric state of the enzymes

The apparent molecular masses of *Lb*Xyn43B and *Lb*Xyn43B /Thr274A were first estimated to 248 and 182 kDa, respectively, by size exclusion chromatography (SEC). These results correspond to a tetramer for *Lb*Xyn43B (monomer 63.58 kDa) and a trimer for *Lb*Xyn43B/Thr274Ala (monomer 63.55 kDa) (Data S1). Thus, mutation to Ala at position 274 appeared to affect the stability of the oligomeric organization of *Lb*Xyn43B. A trimer was, however, unexpected, as previously characterized enzymes were reported either as tetramers or as a dimer, which was reported for the close homolog XynB2 from strain DSM20054, where SEC resulted in an apparent molecular mass of 140 kDa (Michlmayr et al., 2013). In XynB2, the loop in the oligomeric interface holds Ala in position 274, analogous to the mutant *Lb*Xyn43B/Thr274Ala.

To obtain more detailed data, further analysis was made by multi‐detection size exclusion chromatography (OMNISEC). The OMNISEC analyses of *Lb*Xyn43B and *Lb*Xyn43B/Thr274A confirmed that both proteins predominantly existed as tetramers in solution (Table 2) (Data S2). Although the SEC initially suggested a lower molecular weight for the *Lb*Xyn43B/Thr274A consistent with a trimer, the OMNISEC analysis confirmed that the mutant primarily exists as a tetramer (91.3%), but to a reduced hydrodynamic radius (Rhw 4.91 vs. 5.31 nm for *Lb*Xyn43B wt), and reduced intrinsic viscosity (IVw) indicating a more compact conformation. These results suggest that the Thr274Ala substitution enhanced tetramer stability and reduced conformational flexibility without disrupting the tetrameric structure.

**TABLE 2:** OMNISEC analysis of oligomerization state of *Lb*Xyn43B and *Lb*Xyn43B/Thr274Ala.

Parameter	*Lb*Xyn43B		*Lb*Xyn43B/Thr274Ala	
	Peak 1	Peak 2	Peak 1	Peak 2
RV (mL)	15.22	16.48	15.32	16.61
*M*_*w*_ (g/mol)	261,510	202,331	250,731	193,852
*M*_*p*_ (g/mol)	260,290	195,104	260,197	188,311
Calculated *M*_*w*_ (g/mol)	63,580	63,580	63,550	63,550
*M*_*w*_/*M*_*n*_	1.001	1.002	1.001	1.001
IVw (dL/g)	0.0367	0.0362	0.0288	0.0309
Rhw (mn)	5.31	4.6	4.91	4.54
Fraction of sample (%)	84.9	15.1	91.3	8.7
Concentration (g/L)	1.3	1.3	1.3	1.3
Recovery (%)	28.4	28.4	27.7	27.7

Abbreviations: IVw, weight average intrinsic viscosity; *M*_*p*_, molecular weight at the peak apex; *M*_*w*_, average molecular weight; *M*_*w*_/*M*_*n*_, dispersity; Rhw, weight average hydrodynamic radius; RV, retention volume at the peak maximum.

### 3.5 Crystal structure of the β‐d‐xylosidase *Lb*Xyn43B

To further analyze the structure, the enzyme was crystallized and the phase problem of *Lb*Xyn43B was solved by molecular replacement with a resolution of 1.9 Å (Table 3). Clear electron density was observed from Pro6 to Asp540. Consequently, the initial five N‐terminal residues encoded by the natural gene (Met‐Lys‐Ile‐Gln‐Asn) were left unmodeled, and the numbering excluded the N‐terminal histidine tag (Met‐Gly‐Ser‐Ser‐His‐His‐His‐His‐His‐His‐His‐Ser‐Ser‐Gly‐Leu‐Val‐Pro‐Arg‐Gly‐Ser‐His), encoded by the expression vector. The crystal structure, compared with the tetramer AlphaFold model (Figure 1b), showed an RMSD of 0.280 Å across Cα atoms, indicating good structural agreement.

**TABLE 3:** Data collection and refinement statistics of the crystal structure of *Lb*Xyl43B.

Structure	*Lb*Xyl43B
PDB ID	9HE8
Beamline	MAX II, I911‐3
Wavelength (Å)	1.0000
Resolution range	47.0–1.9
Space group	P 1 21 1
Unit cell (Å, °)	78.4, 177.2, 78.8, 90, 98.8, 90
Total reflections	548,439 (16,853)
Unique reflections	163,415
Multiplicity	3.4 (2.3)
Completeness (%)	98.1 (87.5)
Mean *I*/σ(*I*)	10.6 (1.2)
Wilson *B*‐factor (Å^2^)	12.8
*R*_merge_ (*I*)	0.153 (0.898)
*R*_pim_ (*I*)	0.098 (0.717)
CC1/2 (*I*)	0.989 (0.578)
Resolution range for refinement	47.0–1.90 (1.95–1.90)
No. of reflections used in refinement	163,323
No. of reflections in *R*_free_ set	8167
*R*_work_ (F)	0.214
*R*_free_ (F)	0.241
No. of non‐H atoms	18,316
Macromolecules	17,475
Ligands	0
Solvent	827 waters (sum of chains A–D)
Protein residues	540 (for each chain, total 4 chains)
RMSD bonds (Å)	0.013
RMSD angles (°)	1.71
Ramachandran favored (%)	95
Ramachandran allowed (%)	4
Ramachandran outliers (%)	1
Rotamer outliers (%)	2.8
Clashscore	2
Average *B*‐factor (Å^2^)	18.0
No. of TLS groups	4

**FIGURE 4:**
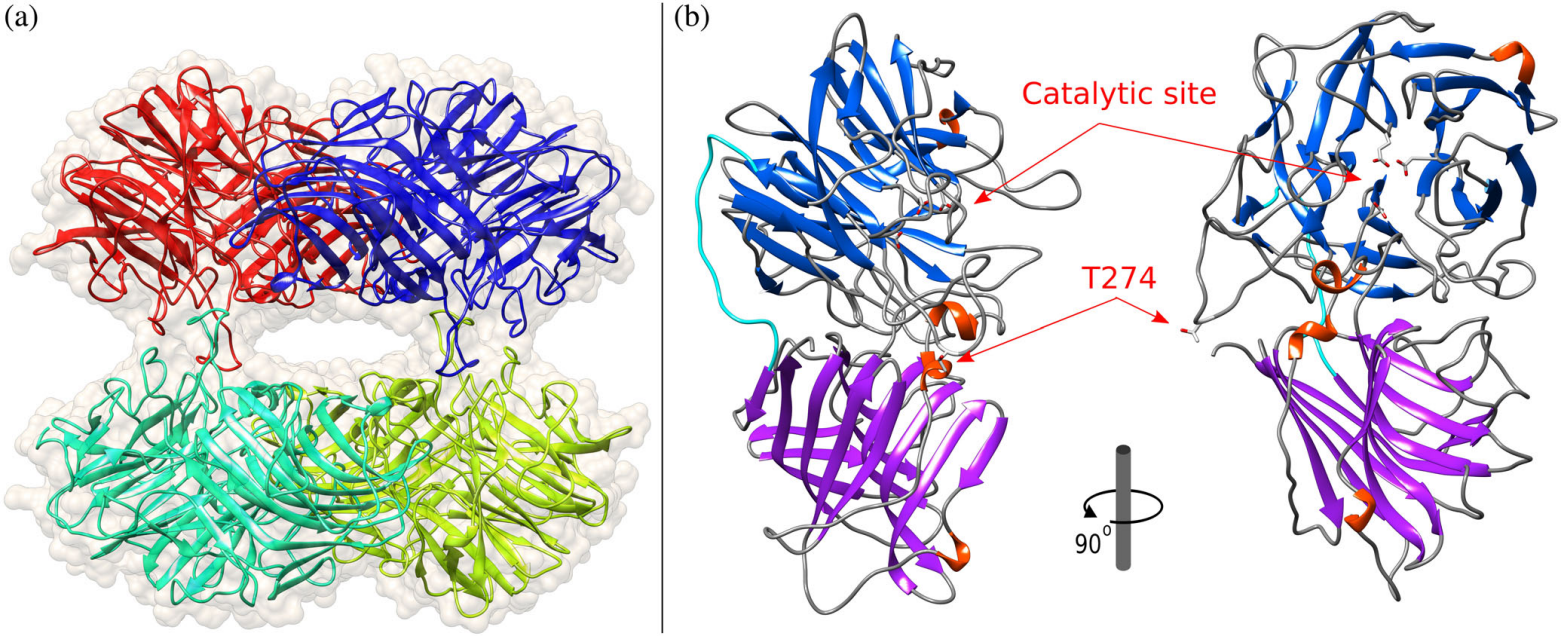
Structure of the β‐xylosidase from *L. brevis* DSM 1269. (a) A cartoon representation of the tetramer with the monomers represented in different colors and coated with a semi‐transparent molecular surface. (b) Details of each monomer showing the N‐terminal catalytic domain with the β‐sheets in blue, the C‐terminal β‐sandwich domain with the β‐sheets in purple and the loop which links both domains in cyan. The side chains of the catalytic residues (Asp14, Asp127, and Glu187) and of the mutated residue T274 are indicated.

*Lb*Xyn43B crystallized in a tetrameric form (Figure 4a), in accordance with the size estimated by SEC and OMNISEC on the native enzyme (above) and with the oligomeric state reported for most xylosidases from GH43, subfamily 11. Each subunit consists of two domains (Figure 4b). The catalytic N‐terminal domain (Pro6‐Asp317) folds into a 5‐bladed β‐propeller structure characteristic of the glycoside hydrolase family 43. The catalytic domain is joined to the C‐terminal β‐sandwich domain (Gln333‐Asp540) by a loop (Ala318‐Ser332).

The tetrameric structure is formed by a dimer of dimers, as is observed in other β‐xylosidases in GH43 (Brüx et al., 2006) (Figure 4a). Each dimer is stabilized in an antiparallel way where one dimer's catalytic domains interact with the other's β‐sandwich domains. Two loops from the catalytic domain, Lys92‐Asp100 (*Lb*Xyn43B numbering, between β‐strands 6 and 7, blade II) and Thr144‐Gly156 (between β‐strands 10 and 11, blade III), interact with the β‐sandwich domain of the other subunit. The sequence of these loops is conserved between the enzymes in the different strains of *L. brevis*. Both dimers stabilize the tetramer through loops in the catalytic domain, creating a large cavity between the dimers exposed to the solvent. The most extended loop that connects β‐strands 16 and 17 in blade V (Ser269‐Arg290 in *Lb*Xyn43B) contains Thr274 (Figure 4b), which is non‐conserved in enzymes from the different strains of *L. brevis*. These differences seem to affect the shape of the tetramer, based on the difference in the apparent native molecular weight between *Lb*Xyn43B and *Lb*Xyn43B/Thr274Ala calculated after SEC and OMNISEC, and may have affected the interpretation of the oligomeric state of XynB2 (Michlmayr et al., 2013), which was estimated as dimeric by SEC.

### 3.6 Molecular dynamics simulations

**FIGURE 5:**
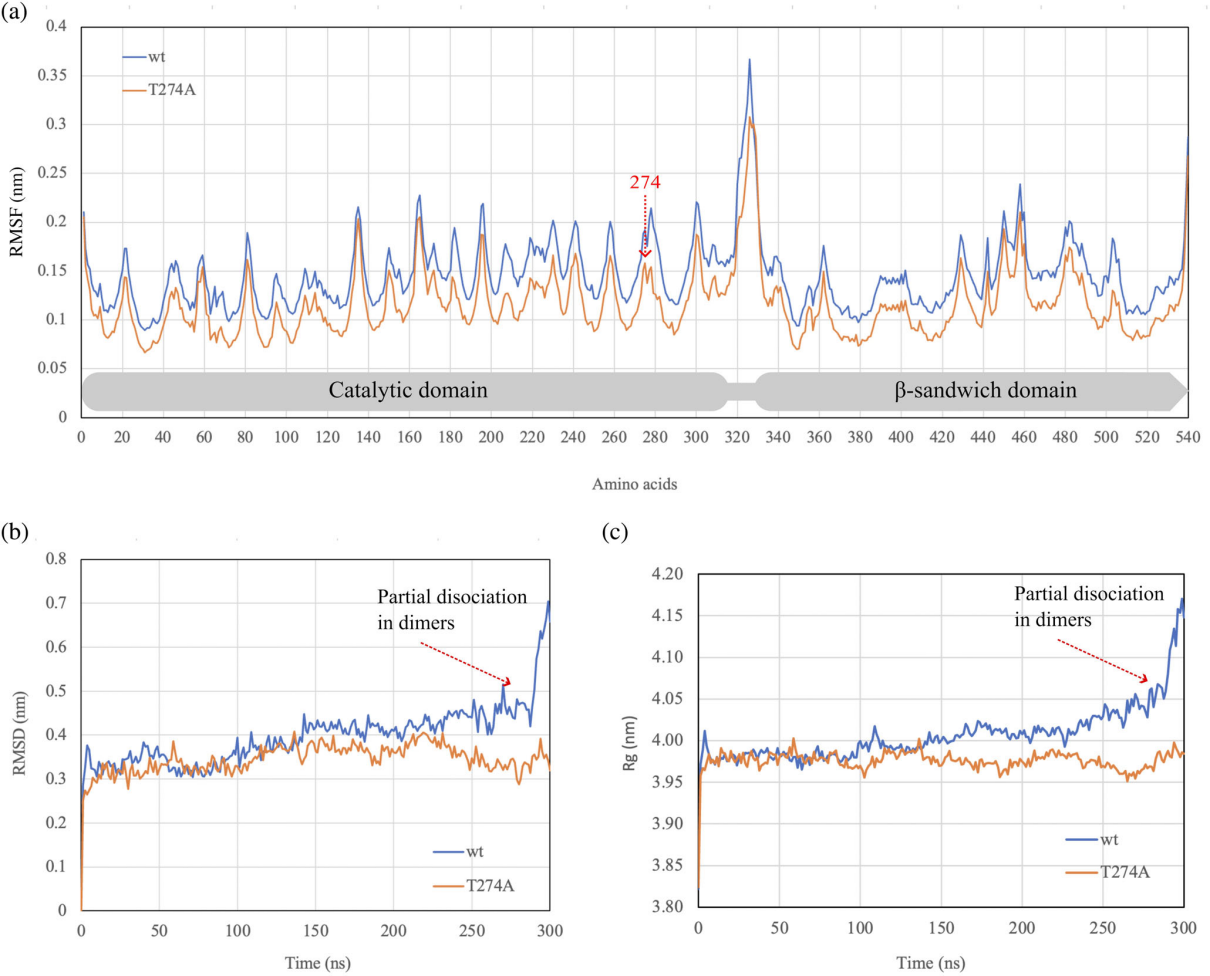
Molecular dynamics simulations of the tetramers of *Lb*Xyn43B (blue) from strain DSM1269 and its mutant Thr274Ala (red). All data plotted correspond to the average of triplicates. (a) Root mean square fluctuations (RMSF). (b) Root mean square deviations (RMSD) of *C*_*α*_ atoms. (c) Radius of gyration (*R*_*g*_).

To further analyze potential effects on the shape of the respective protein, the tetrameric structure of *Lb*Xyn43B wt and the modeled tetrameric structure of *Lb*Xyn43B/Thr274Ala were subjected to molecular dynamics simulations for 300 ns. The RMSFs (Figure 5a) are similar in both proteins, reaching their highest values in the amino acids located in the loop that links the two domains, suggesting their high flexibility. However, this loop is oriented to the solvent and is not involved in the oligomeric interactions.

The RMSDs for *C* *_α_* from the energetically minimized starting structures showed differences between the two proteins (Figure 5b). *Lb*Xyn43B reached the first equilibrium stage from 5 to 100 ns, with the RMSD fluctuating between 0.3 and 0.4 nm. Later, it increased into a range of 0.4–0.5 nm, from 150 to 280 ns. After this, the RMSD rose dramatically to 0.7 nm at the end of the simulation, at 300 ns. This significant change is attributed to the partial dissociation of *Lb*Xyn43B wt into dimers. On the other hand, the RMSD for *Lb*Xyn43B/Thr274Ala remained stable from 10 to 300 ns, fluctuating from 0.3 to 0.4 nm, and no dissociation was observed. These results are consistent with the radius of gyration (*R*_*g*_) (Figure 5c), which increases from an average of 4 nm at 100 ns to 4.15 nm at 300 ns in *Lb*Xyn43B. In contrast, the *R*_*g*_ of *Lb*Xyn43B/Thr274Ala remains stable throughout the simulation, with an average value of 3.9 nm (5–300 ns). Thus, the MD simulations indicated that the mutation Thr274Ala maintains the protein in a compacted state while the wt partially dissociates.

**FIGURE 6:**
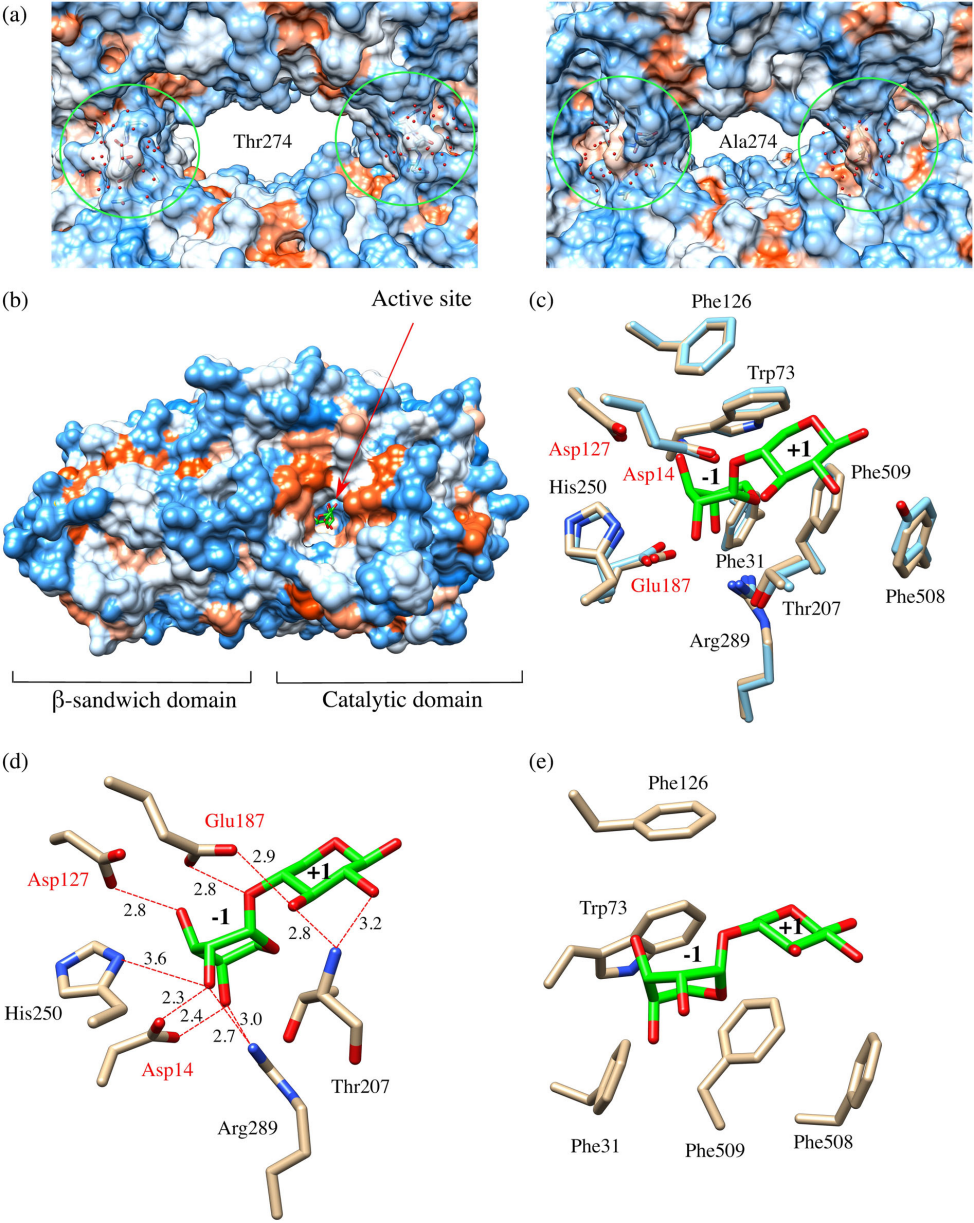
Central cavity of the tetrameric structure and active site of the β‐xylosidase from *L. brevis* DSM 1269 (*Lb*Xyn43B). (a) Central cavity of *Lb*Xyn43B wt and mutant Thr274Ala (MD snapshot at 200 ns) represented in hydrophobicity surface according to the Kyte‐Doolittle scale (Kyte & Doolittle, 1982), from blue for the most hydrophilic, through white to orange‐red for the most hydrophobic. (b) Active site in a single subunit. The ligand xylobiose was transferred from the co‐crystal structure of a mutated xylanase from *Bacillus pumilus* IPO (*Bp*XynB) (PDB: 5ZQS) (Hong et al., 2018). (c) Superimposition of the catalytic site amino acids from *Lb*Xyn43B and complex xylobiose/*Bp*XynB. Glu187 and Phe508 from *Lb*Xyn43B correspond to Gln186 and Tyr503 in *Bp*XynB‐E186Q/F503Y. (d) Predicted hydrogen‐bonding interactions between the catalytic triad and the substrate are shown in red, with distances shown in Å. (e) Predicted hydrophobic interactions.

All these results are consistent with the experimental findings. OMNISEC analysis showed that the tetrameric *Lb*Xyn43B/Thr274Ala was more compact than the *Lb*Xyn43B wt. In addition, the thermal stability analysis revealed a slightly higher *T*_*m*_ for the *Lb*Xyn43B/Thr274Ala than for *Lb*Xyn43B wt, which can be attributed to the more compact structure of the mutated variant. The replacement of Thr274 with Ala in the loop at the subunit interface in *Lb*Xyn43B thus promoted a more compact tetrameric structure by reducing steric hindrance and enhancing hydrophobic interactions at the oligomeric interface (Figure 6a). Whether a similar compact structure is the reason for the interpretation that XynB2 is dimeric remains to be investigated, as there currently is only SEC data available for this enzyme.

### 3.7 Catalytic site of *Lb*Xyn43B

GH43 enzymes catalyze substrate hydrolysis with inversion of the configuration of the anomeric carbon (Pitson et al., 1996) and a triad of catalytic residues has been proposed (Nurizzo et al., 2002). The three catalytic residues are highly conserved in GH43 family members whose structure has been determined, also including other subfamilies than 11, for example, subfamily 26 (Linares‐Pastén et al., 2017).

The active site of *Lb*Xyn43B is in the center of the catalytic domain, is solvent accessible to allow substrate access, and is orientated towards the central cavity of the tetrameric oligomer (Figure 6a,b). The catalytic amino acids are very well conserved (Figure 6c), allowing unambiguous identification of Glu187 as the proton donor, Asp14 as the catalytic nucleophile/base, and Asp127 as the auxiliary residue. Glu187 is positioned above the glycosidic bond, which is consistent with its catalytic role (Figure 6d). To analyze enzyme/substrate interactions in *Lb*Xyn43B, the crystal structure was superimposed on that of a co‐crystallized xylobiose/*Bp*XynB‐E186Q/F503Y structure from *Bacillus pumilis* (Hong et al., 2018) (Figure 6c). Prior to the introduction of the two active site mutations (Glu186Gln and Phe503Tyr), this enzyme shared 49.2% sequence identity with *Lb*Xyn43B.

The enzyme *Lb*Xyn43B prefers small XOS substrates (Table 4) and has two sub‐sites (−1 and +1) as previously proposed for enzymes in GH43 subfamily 11 (Hong et al., 2018). However, kinetic analysis of, for example, WXyn43 from *Weissella cibaria* suggests that further interactions may occur in GH43 subfamily 11 enzymes that either could be due to stabilization of the substrate due to H‐bonding or other interactions in a potential +2 subsite (Falck et al., 2015). By superimposition of the *Bp*XynB‐Glu186Gln/Phe503Tyr structure, including the xylobiose ligand, sub‐sites −1 and +1 were identified. Sub‐site −1 has most of the hydrogen bond interactions with the ligand, including with the catalytic residues Asp14 and Asp127, as well as with Arg289 and His250 (Figure 6d) and hydrophobic interactions with Phe31, Trp73, Phe126, and Phe509 (Figure 6e). In sub‐site +1, there is a predominance of hydrophobic interactions, and both Phe508 and Phe509 are in direct contact with the ligand (Figure 6e). Still, both Glu187 and the backbone of Thr207 can participate in hydrogen bonding interactions with the substrate in the +1 subsite (Figure 6d). These substrate‐interacting residues from the −1 and +1 subsites are also completely conserved in the WXyn43 sequence, indicating that the higher catalytic efficiency for this enzyme must be dependent on further interactions with less conserved residues.

**TABLE 4:** Specific activity and kinetic analysis of wild‐type *Lb*Xyn43B and the Thr274Ala variant on natural substrates in comparison with taxonomically related enzymes from the same subfamily (GH43_11), including XynB2 from *L. brevis* DSM 20054, *Lb*X from *L. brevis* ATCC367, and WXyn43 from *Weissella cibaria* strain 92.

Enzyme	Substrate	Spec. activity (U/mg)	*k*_cat_ (s^−1^)	*K*_m_ (mM)	*k*_cat_/*K*_m_ (mM^−1^ s^−1^)	References
*Lb*Xyn43B^(a)^	β‐1,4‐d‐xylobiose	215 ± 11	349.6 ± 48.3	8.9 ± 3	41.2 ± 8.4	Present work
	β‐1,4‐d‐xylotriose	173 ± 20	247.2 ± 16.1	6.5 ± 1.1	36.6 ± 4.9	
	β‐1,4‐d‐xylotetraose	107 ± 9	150.0 ± 8.8	4.5 ± 0.7	33.5 ± 5.5	
*Lb*Xyn43B‐Thr274Ala^(a)^	β‐1,4‐d‐xylobiose	161 ± 18	317.4 ± 24.8	11.7 ± 2.2	27.4 ± 3	Present work
	β‐1,4‐d‐xylotriose	131 ± 6.5	199.8 ± 50.2	6.1 ± 2.9	35.3 ± 7.5	
	β‐1,4‐d‐xylotetraose	102 ± 5	127.6 ± 6.8	3.6 ± 1.1	38.6 ± 5.5	
*W*Xyn43^(b)^	β‐1,4‐d‐xylobiose	100 ± 5	961.0 ± 25	7.2 ± 0.5	133.5 ± 9.9	Falck et al. (2015)
	β‐1,4‐d‐xylotriose	98 ± 4	900.0 ± 13	6.5 ± 0.3	138.5 ± 6.7	
	β‐1,4‐d‐xylotetraose	29 ± 2	770.0 ± 7	17 ± 0.3	45.3 ± 0.9	
XynB2^(c)^	β‐1,4‐d‐xylobiose	154 ± 6	233	4.8 ± 0.4	48	Michlmayr et al. (2013)
*Lb*X^(d)^	β‐1,4‐d‐xylobiose		407.0 ± 9	2.96 ± 0.24	138 ± 9	Jordan et al. (2013)
	β‐1,4‐d‐xylotriose		235.0 ± 4	2.91 ± 0.15	80.8 ± 3.0	
	β‐1,4‐d‐xylotetraose		146.0 ± 4	2.40 ± 0.08	32.6 ± 1.2	

*Note*: Reactions performed at ^(a)^37°C and pH 7.0, ^(b)^37°C and pH 6.0, ^(c)^37°C and pH 5.5, and ^(d)^25°C and pH 6.0.

### 3.8 Kinetic characterization

The kinetic constants (Table 4) were statistically analyzed with the Student *t*‐test to verify any significant difference. The analysis showed that the *k*_cat_ for X4 differed significantly between *Lb*Xyn43 and the variant *Lb*Xyn43B‐Thr274Ala, while this was not the case for the shorter substrates (X2 and X3). Comparison of the activity of each individual enzyme on the different substrates (X2–X4) showed that the *k*_cat_ for *Lb*Xyn43 was significantly higher for the shortest substrate (X2) and decreased with increasing substrate length. For the variant *Lb*Xyn43B‐Thr274Ala, there was a similar trend, but in this case, the difference between the *k*_cat_ for X3 and X4 was not statistically significant. The *K*_m_ was not significantly different between the enzymes, and not for the different XOS substrates (X2–X4) in a single enzyme, except for the difference in *K*_m_ between X2 and X4 in the *Lb*Xyn43B‐Thr274Ala variant (Table 4; Data S3).

Although the differences between the wt and the Thr247A variant are limited, they may be attributed to a minor change in binding affinity due to structural rearrangements that result from the change in the shape of the oligomeric enzyme. The observed variations in *k*_cat_ values indicate differences in enzyme turnover rates across substrates that may be influenced by accessibility and are typical of GH43 subfamily 11, while more consistent *K*_m_ values suggest stable substrate binding affinities. However, the exception in *Lb*Xyn43B‐Thr274Ala, where a significant difference in *K*_m_ between X2 and X4 is found, suggests a distinct binding affinity that could involve structural rearrangement specific to this enzyme‐substrate interaction.

The catalytic efficiencies towards X2 reported here for both *Lb*Xyn43B and its variant Thr274Ala are close to the corresponding values for X2 reported for XynB2 from *L. brevis* DSM20054 (Michlmayr et al., 2013), although the latter was analyzed at a different pH (see Table 4) Unfortunately, no data is available for X3 or X4 for XynB2, making further comparisons difficult. Catalytic efficiencies on X2 to X4 have, however, been reported for the xylosidase *Lb*X from *L. brevis* ATCC367 (Table 4), which differs from *Lb*Xyn43B only in residues 195 (Ser/Asn) and 460 (Asn/Asp; while both enzymes have Thr at position 274) and follows the trend of decreased catalytic efficiency with increased degree of polymerization (DP) of the substrate at the respective condition used. The *K*_m_ did not decrease with increasing DP, which indicates no further interactions beyond subsite +1. It should, however, be noted that the data for LbX were also collected at a lower temperature (Table 4), which may, for example, influence the binding of the substrate. Also, the oligomeric state for *Lb*X has not been determined, and it is not yet known whether it is tetrameric. Significantly higher catalytic efficiency was reported for the tetrameric enzyme WXyn43 from the taxonomically related *Weissella cibaria* strain 92 (Table 2). The high catalytic efficiency measured for WXyn43 may, however, be due to differences in interactions close to the active site, as the sequence conservation between WXyn43 and *Lb*Xyn43A is lower (76.8% ID).

## 4 DISCUSSION

To date, annotated genomic data is available for several *L. brevis* strains, and gene analysis shows that each of the strains encodes two enzymes of GH43 subfamily 11 (xylosidases) and subfamily 26 (arabinofuranosidases), respectively. We have previously solved the structure of the arabinofuranosidase from *L. brevis* strain DSM1269 (Linares‐Pastén et al., 2017) and modeled the structure of the xylosidase from a *Weissella* strain (Falck et al., 2015), later identified as *W. cibaria* strain 92 (Månberger et al., 2020). In this work, we have focused on a gene encoding for the subfamily 11 candidate from the same *L. brevis* strain (DSM1269). According to the CAZy data bank (www.cazy.org) 24 enzymes from GH43 subfamily 11 (19 bacterial and 1 fungal, 2024/06/14) have been characterized, and 7 of the characterized bacterial enzymes have determined structures, including the β‐xylosidases from *Bacillus halodurans* C‐125 (renamed *Halalkalibacterium halodurans* C‐125), two strains of *B. pumilis* (IPO and PLS), *B. subtilis* strain 168, *Enterobacter* sp. culture clone nf1B6, *Geobacillus stearothermophilus* T‐6 NCIMB 40222, and the mixed β‐xylosidase/α‐l‐arabinofuranosidase from *Selenomonas ruminantium* GA192. The *Lb*Xyn43B structure solved in this work is the first from LAB available in subfamily 11.

Crystallographic structural comparison with other GH43 β‐xylosidases of subfamily 11 reveals that the tetrameric assembly is highly conserved (Table 5). Dimeric forms have been observed only in β‐xylosidases from *Bacillus pumilus* (BpX and XynB) and *Halalkalibacterium halodurans* (XynB). Notably, BpX has been crystallized in both dimeric and tetrameric states, and SEC‐MALS analysis confirmed the coexistence of these forms in solution, with ~32% tetramer and ~68% dimer (Hong et al., 2018). In contrast, the dimeric structure of *H. halodurans* XynB represents a distinct quaternary arrangement and is characterized by a shortened inter‐subunit loop, which may underlie its inability to form a tetramer. Despite these differences, both oligomeric states across the subfamily can accommodate ligand binding, as evidenced by the co‐crystal structures of XynB3 from *G. stearothermophilus* and both the tetrameric and dimeric forms of BpX from *B. pumilus*.

**TABLE 5:** Crystal structures of b‐xylosidases, family GH43, subfamily 11.

Enzyme	Source	PDB code	Ligand	Oligomer	Resolution (Å)
β‐xylosidase (*Lb*Xyn43B)^a^	*Levilactobacillus brevis* DSM1269	9HE8 [A,B,C,D]		Tetramer	1.9
β‐1,4‐xylosidase (BsX; XynB; YnaK; BSU17580)	*Bacillus subtilis* subsp. *subtilis* str. 168	1YIF [A,B,C,D]		Tetramer	1.8
β‐xylosidase (XynD;CAC3452)	*Clostridium acetobutylicum* ATCC 824	1Y7B [A,B,C,D]		Tetramer	1.6
		1YI7 [A,B,C,D]		Tetramer	1.9
β‐xylosidase (XynB3;HUS_CDS105)	*Geobacillus stearothermophilus* T‐6 NCIMB 40222	2EXH [A,B,C,D]		Tetramer	1.88
		2EXI [A,B,C,D]		Tetramer	2.15
		2EXJ [A,B,C,D]	β‐d‐Xylp‐(1–4)‐β‐d‐Xylp	Tetramer	2.2
		2EXK [A,B,C,D]	β‐d‐Xylp‐(1–4)‐β‐d‐Xylp	Tetramer	2.2
β‐xylosidase/α‐l‐arabinofuranosidase (Xsa; Sxa; SXA; SrXyl43)	*Selenomonas ruminantium* GA192	3C2U [A,B,C,D]		Tetramer	1.3
β‐xylosidase (Xyl43; EcXyl43)	*Enterobacter* sp. enrichment culture clone nf1B6	7K1R [A,B,C,D]		Tetramer	2.4
		8UWS [A,B,C,D]		Tetramer	2.65
β‐xylosidase (BpX; XynB; E‐BXSEBP)	*Bacillus pumilus* IPO	5ZQX [A,B,C,D]	β‐d‐Xylp‐(1–4)‐β‐d‐Xylp	Tetramer	2.0
		5ZQS [A,B]	β‐d‐Xylp β‐d‐Xylp‐(1–4)‐β‐d‐Xylp	Dimer	1.78
		5ZQJ [A,B]		Dimer	1.73
β‐xylosidase (XynB)	*Bacillus pumilus* PLS	6IFE [A,B]		Dimer	1.8
β‐xylosidase (XynB; XylBH43; BH3683)	*Halalkalibacterium halodurans* C‐125	1YRZ [A,B]		Dimer	2.0

^a^ Structure solved in this work.

The closest LAB β‐xylosidase to *Lb*Xyn43B is the homology model of the GH43 subfamily 11 enzymes from *W. cibaria* strain 92 (Falck et al., 2015), which is highly active with high *k*_cat_ (Table 4). Kinetic studies on the XOS substrates showed that the turnover (*k*_cat_) and catalytic efficiency (*k*_cat_/*K*_m_) values of *Lb*Xyn43B are higher for the shortest chain length XOS. This is like most other reported β‐xylosidases from GH43_11 (Table 4). The turnover number (*k*_cat_) of both *Lb*Xyn43B and *Lb*Xyn43B/Thr274Ala was higher for X2 than for X3, while the *K*_m_ showed a decreasing trend (although not statistically significant) for X3 compared with X2, indicating that this enzyme may have some further substrate interactions in addition to the two subsites (−1 and +1), shown for other structurally studied GH43 subfamily 11 β‐xylosidases. The superimposition of X2 bound in the active site of the *B. pumilis* enzyme clearly shows that the −1 and +1 subsites are very well conserved. However, some additional interactions may occur, as the *K*_m_ was lowest using the X3 substrate. This may be a result of interdomain interactions, as the mutated variant (*Lb*Xyn43B/Thr274Ala) displayed a weak opposite trend in catalytic efficiency related to oligosaccharide length compared with the wt, which may be due to the minor structural rearrangements of the tetramer observed by molecular dynamics.

Growth trials using XOS substrates as carbon sources for *L. brevis* DSM1269 showed that xylobiose and xylotriose are both well utilized by the bacterium (Faryar et al., 2015), supporting that these oligosaccharides are taken up and are the primary substrates for the intracellular *Lb*Xyn43B. Gene cluster analyses in other *Levilactobacillus* spp. have shown that a three‐component system consisting of a transcriptional regulator, a transporter, and glycoside hydrolase(s) can be sufficient for the utilization of potential prebiotics, irrespective of the type of transporter identified (i.e., ATP‐binding cassette (ABC) transporter family, glycoside‐pentoside‐hexuronide (GPH): cation symporter family, or permease of the phosphotransferase system (PTS)), as exemplified in the analysis of prebiotics uptake in *Lactobacillus acidophilus* NCFM (Andersen et al., 2012; Quistgaard et al., 2016). The transporters in *L. acidophilus* NCFM showed different efficiency in uptake, which was dependent on the length of the oligosaccharides, with PTS permeases having higher selectivity towards disaccharides. In contrast, ABC systems appeared to be also induced by the longer oligosaccharides. Which system is dominant in the uptake of XOS in *L. brevis* DSM1269 has not yet been investigated. However, the gene encoding the homolog *Lb*X in *Levilactobacillus brevis* ATCC 367 resides in a putative XOS utilization locus that encompasses an AraC transcriptional regulator and a predicted GPH transport system, consistent with the higher efficiency of the enzyme on smaller substrates.

The mutagenesis of Thr274 to Ala resulted in an apparent decrease in molecular weight, in line with the observation after SEC for XynB2 from the strain DSM20054 (Michlmayr et al., 2013). However, the OMNISEC analysis and molecular dynamics simulations of the respective structure studied in this work showed that the explanation for this apparent lower molecular weight is a change in shape and not in oligomerization, where the mutated enzyme adopts a more compact oligomeric form. Such changes in shape, occurring as in this case after a single mutation far away from the active site, may still affect the accessibility or binding of the substrates. The differences in catalytic efficiency between the studied xylosidases of GH43 subfamily 11 may thus not only be a consequence of direct interactions in the active site subsites but that the changes in shape affect interdomain interactions despite maintaining a similar degree of oligomerization. Nevertheless, the changes in catalytic activity were rather small, and beyond its physiological role in xylooligosaccharide catabolism, *Lb*Xyn43B represents a valuable model system for investigating oligomerization dynamics and loop‐mediated structural modulation. The clear impact of a single loop residue (Thr274) on quaternary structure, thermostability, and molecular shape, without significantly altering catalytic function, illustrates principles relevant for protein engineering, stability prediction, and computational modeling. Such features position *Lb*Xyn43B as a benchmark system for integrative structural biology combining crystallography, mutagenesis, and MD simulations.

## 5 CONCLUSION

The crystal structure of the β‐1,4‐xylosidase from *Levilactobacillus brevis* DSM1269 has been solved at 1.9 Å resolution, revealing a two‐domain enzyme composed of a catalytic domain with a 5‐fold β‐propeller fold, typical in the family GH43, connected to a C‐terminal β‐sandwich domain. Both crystallographic data and size exclusion chromatography show that the protein has a tetrameric structure and that the shape of the tetramer is dependent on loop interactions affecting the radius of gyration. The kinetic studies showed that the kinetic parameters could be slightly affected by the shape of the oligomer but that a clear preference for xylobiose and xylotriose was kept in line with the proposed intracellular location. The previously shown ability of *L. brevis* DSM1269 to utilize xylobiose and xylotriose as carbon sources for growth, combined with the current data, leads us to propose that *Lb*Xyn43B is the enzyme responsible for the first step in the intracellular metabolism of xylooligosaccharides in *L. brevis* DSM1269. With the growing interest in lignocellulosic materials (e.g., straw and bran) as feedstocks for the manufacture of higher value products, degradation of the oligosaccharides can, apart from being valuable in the intestine, also have industrial interest as the availability of monosaccharides (after *Lb*Xyn43B hydrolysis) opens up possibilities for additional microorganisms to utilize these carbohydrate resources in biorefining applications for, for example, platform chemicals production. Our work also shows that a single mutation may be sufficient in gaining higher thermostability and compactness of oligomeric enzymes, making *Lb*Xyn43B an interesting model for structural studies.

## AUTHOR CONTRIBUTIONS

**Javier A. Linares‐Pastén:** Conceptualization; data curation; formal analysis; visualization; writing – original draft; methodology; investigation; supervision; project administration; writing – review and editing; software; validation; resources; funding acquisition. **Reza Faryar:** Formal analysis; investigation; methodology. **Sergio Torrez Alvarez:** Methodology; investigation; formal analysis. **Khalil Albasri:** Methodology. **Bashar Shuoker:** Methodology. **Maher Abou Hachem:** Supervision; writing – review and editing. **Derek T. Logan:** Formal analysis; data curation; methodology; investigation; supervision; writing – review and editing; software; validation; resources. **Eva Nordberg Karlsson:** Conceptualization; formal analysis; funding acquisition; investigation; project administration; supervision; writing – review and editing; resources; validation.

## CONFLICT OF INTEREST STATEMENT

The authors declare that they have no conflict of interest.

## Supporting information


**Data S1.** Supporting Information.

## Data Availability

The data that support the findings of this study are openly available in [PDB] at [https://doi.org/10.2210/pdb9HE8/pdb], reference number [9HE8].
