# Supplementary material for: A single residue affects the dynamics and shape of a tetrameric GH43 β‐1,4‐d‐xylosidase from Levilactobacillus brevis DSM1269
Source: Protein Sci. 2025 Sep 13;34(10):e70299. doi: 10.1002/pro.70299 (PMC12432433; doi:10.1002/pro.70299)

Supplementary Information

A single residue affects the dynamics and shape of a tetrameric GH43 β-1,4-D-xylosidase from *Levilactobacillus brevis* DSM1269

Javier A. Linares-Pastén [1,*], Reza Faryar [1], Sergio Torrez Alvarez[1,3], Khalil Albasri[1], Bashar Shuoker[1,4], Maher Abou Hachem[4], Derek T. Logan[2], Eva Nordberg Karlsson[1]

[1]Biotechnology and Applied Microbiology, Department of Process and Life Sciences Engineering, LTH, Lund University, PO, P.O Box 124, Lund, Sweden.

[2]Biochemistry and Structural Biology, Department of Chemistry, Lund University, P.O Box 124, Lund, Sweden

[3]Instituto de Investigaciones Químicas IIQ, Universidad Mayor de San Andrés UMSA, Av. Villazón N° 1995, 0201-0220 La Paz, Bolivia.

[4]Department of Biotechnology and Biomedicine, Technical University of Denmark.

*Correspondence: javier.linares_pasten@biotek.lu.se

S1. Size-exclusion chormatogrphy analysis

Standards

Mw (kDa)	LN[MW]	Ve (mL)	V_e/V_o
669	6.5	33.71	1.07
443	6.08	37.17	1.18
200	5.3	41.90	1.33
150	5.01	44.73	1.42
66	4.19	50.09	1.59
29	3.37	59.54	1.89

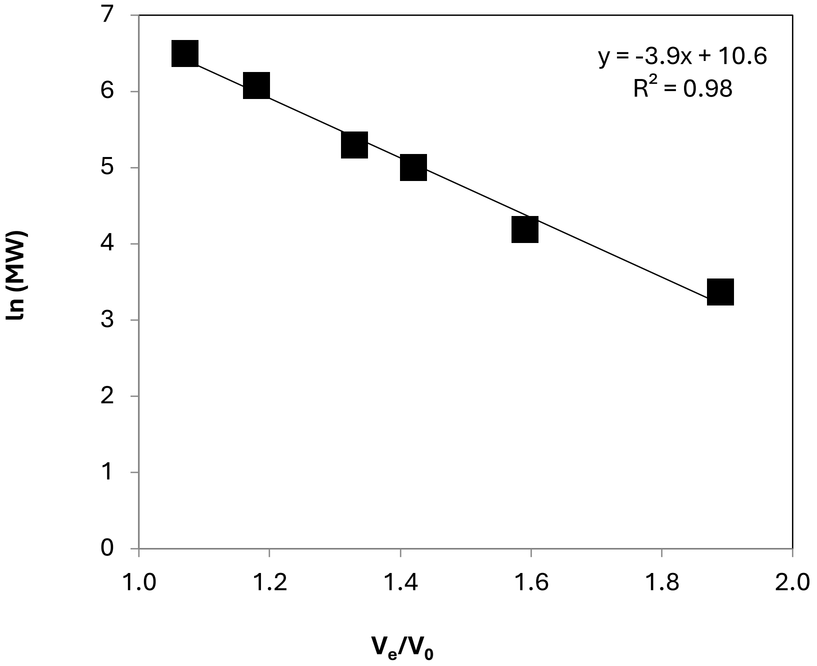

Protein	V_e (mL)	V_0 (mL)	V_e/V_0	ln(MW)	MW (kD)	MW (kD) monomer	N° Subunits
*Lb*Xyn43B	41.0	31.5	1.30	5.52	250.59	63.58	3.9 (~4)
LbXyn43B-T243A	44.1	31.5	1.40	5.14	170.72	63.55	2.7 (~3)

Supplementary Information

S2 OMNISEC analyses

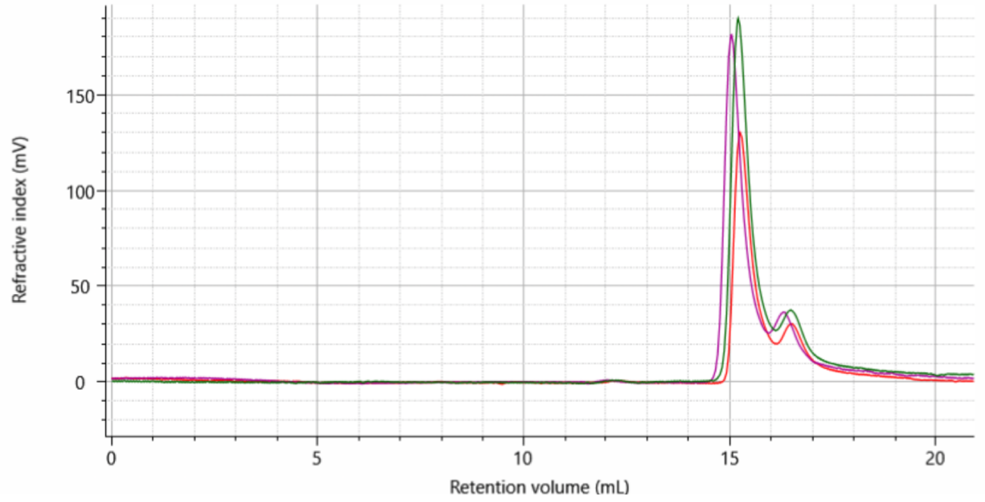

***Lb*Xyn43B.** Raw data overlay, showing refractive index for injection 1 (in red), 2 (in purple) and 3 (in green) plotted in function of retention volume (in mL).

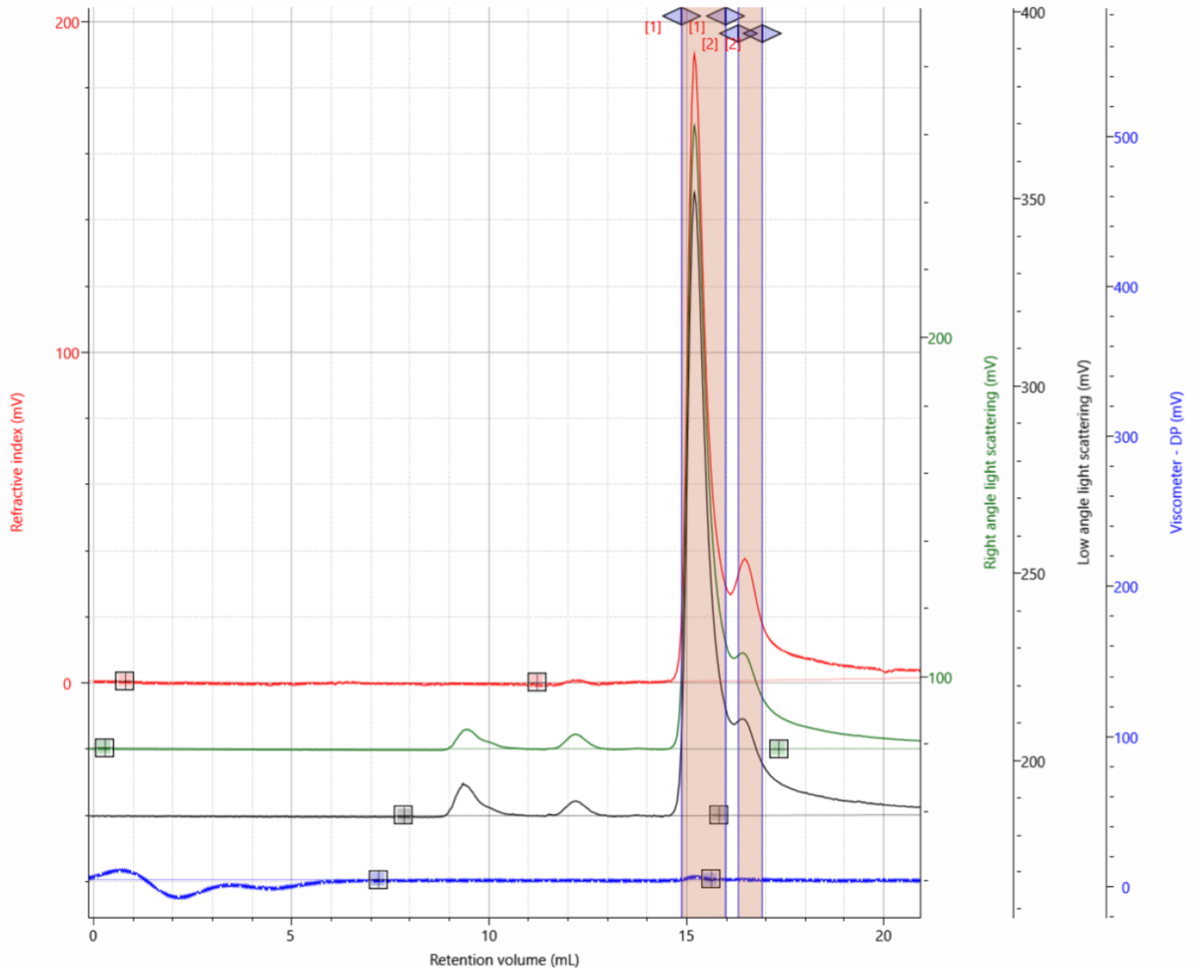

***Lb*Xyn43B.** Raw data of the chosen injection, showing refractive index (in red), right angle light scattering (in green), low angle light scattering (in black) and viscometer (in blue) plotted in function of retention volume (in mL).

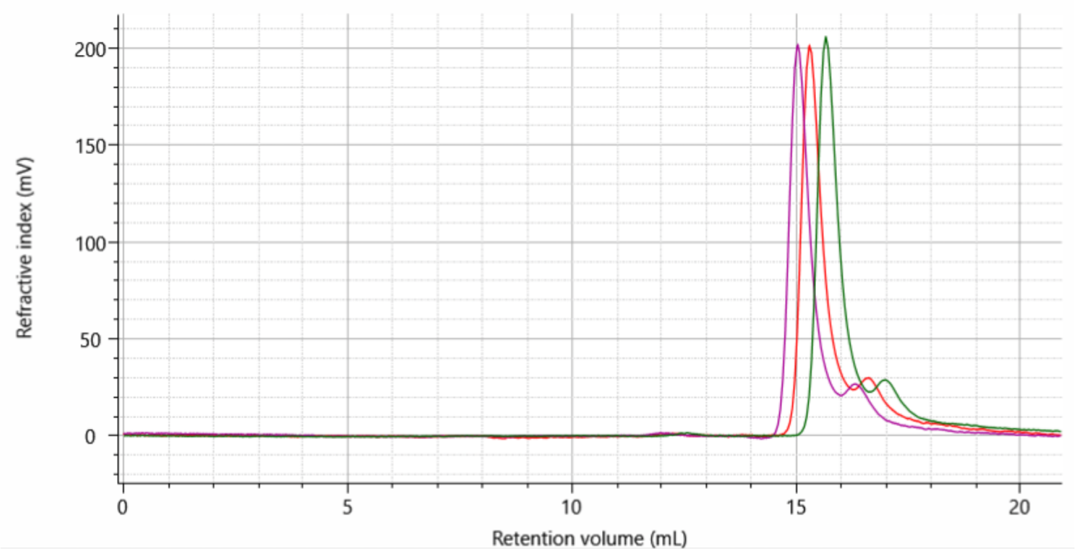

Lb**Xyn43B-T274A.** Raw data overlay, showing refractive index for injection 1 (in red), 2 (in purple) and 3 (in green) plotted in function of retention volume (in mL).

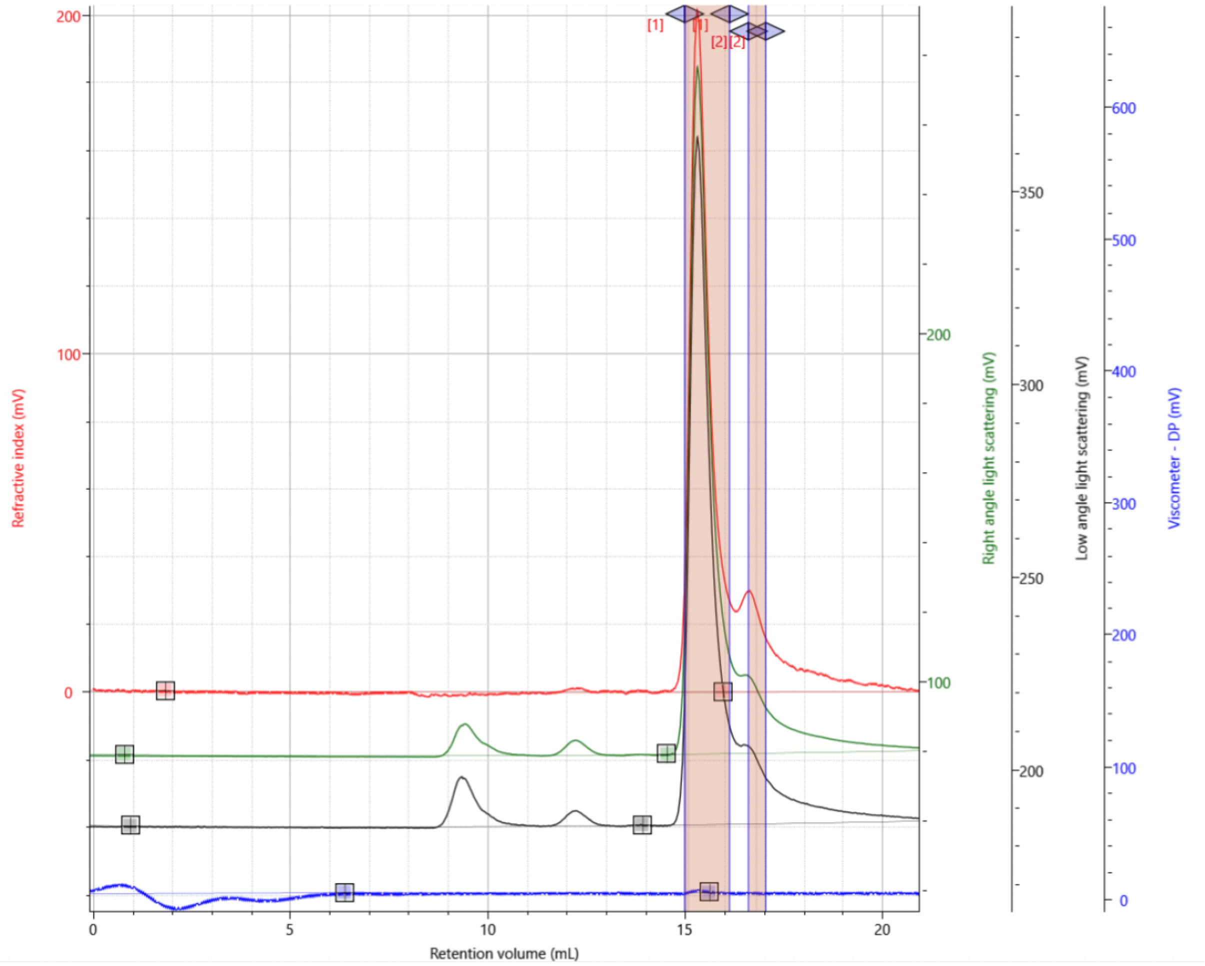

Lb**Xyn43B-T274A.** Raw data of the chosen injection, showing refractive index (in red), right angle light scattering (in green), low angle light scattering (in black) and viscometer (in blue) plotted in function of retention volume (in mL).

Supplementary Information

S3. Enzyme kinetics (See Table 4)

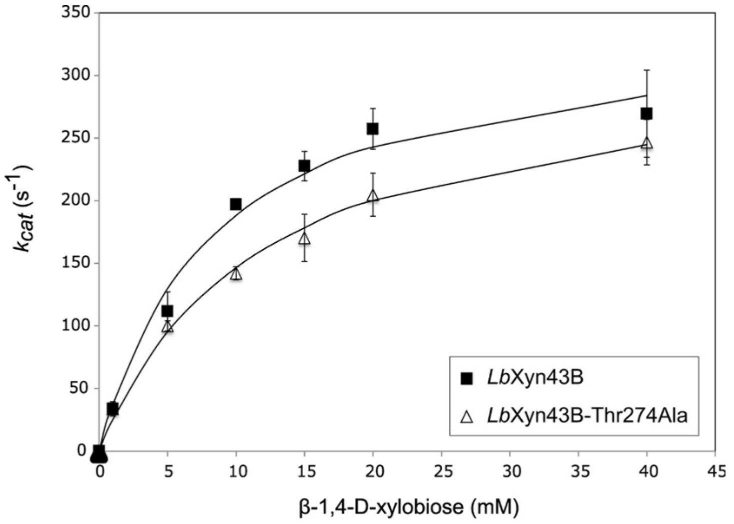

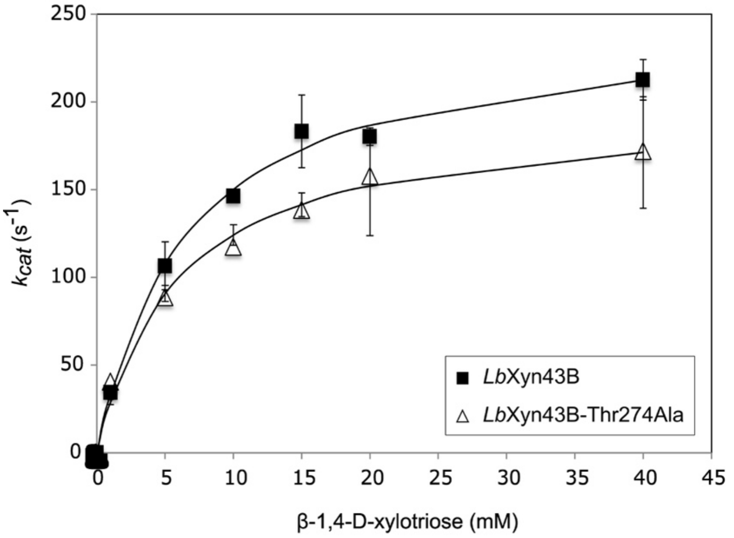

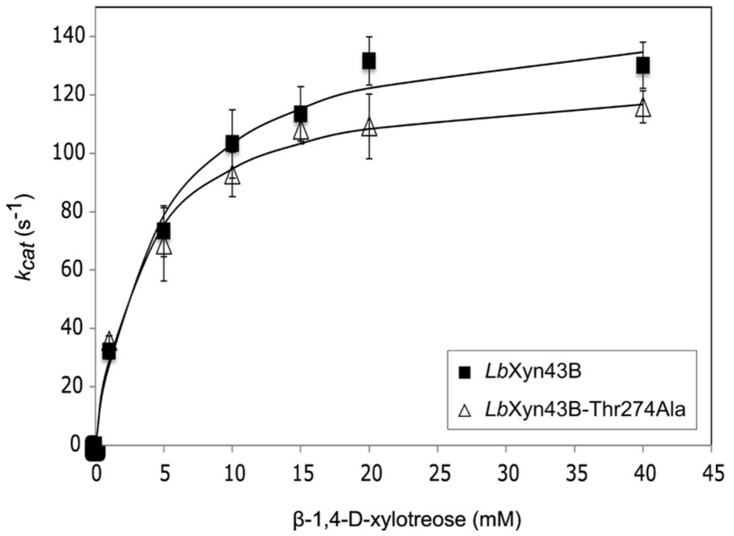

Supplement: Supplementary file 1 — Data S1. Supporting Information. [file PRO-34-e70299-s001.pdf]
